# Supplementary figures and images for: Skeletal ossification of Middle Triassic pachypleurosaur Keichousaurus hui (Reptilia: Sauropterygia) revealed by zinc distribution
Source: PeerJ. 2025 Jun 18;13:e19475. doi: 10.7717/peerj.19475 (PMC12182052; doi:10.7717/peerj.19475)

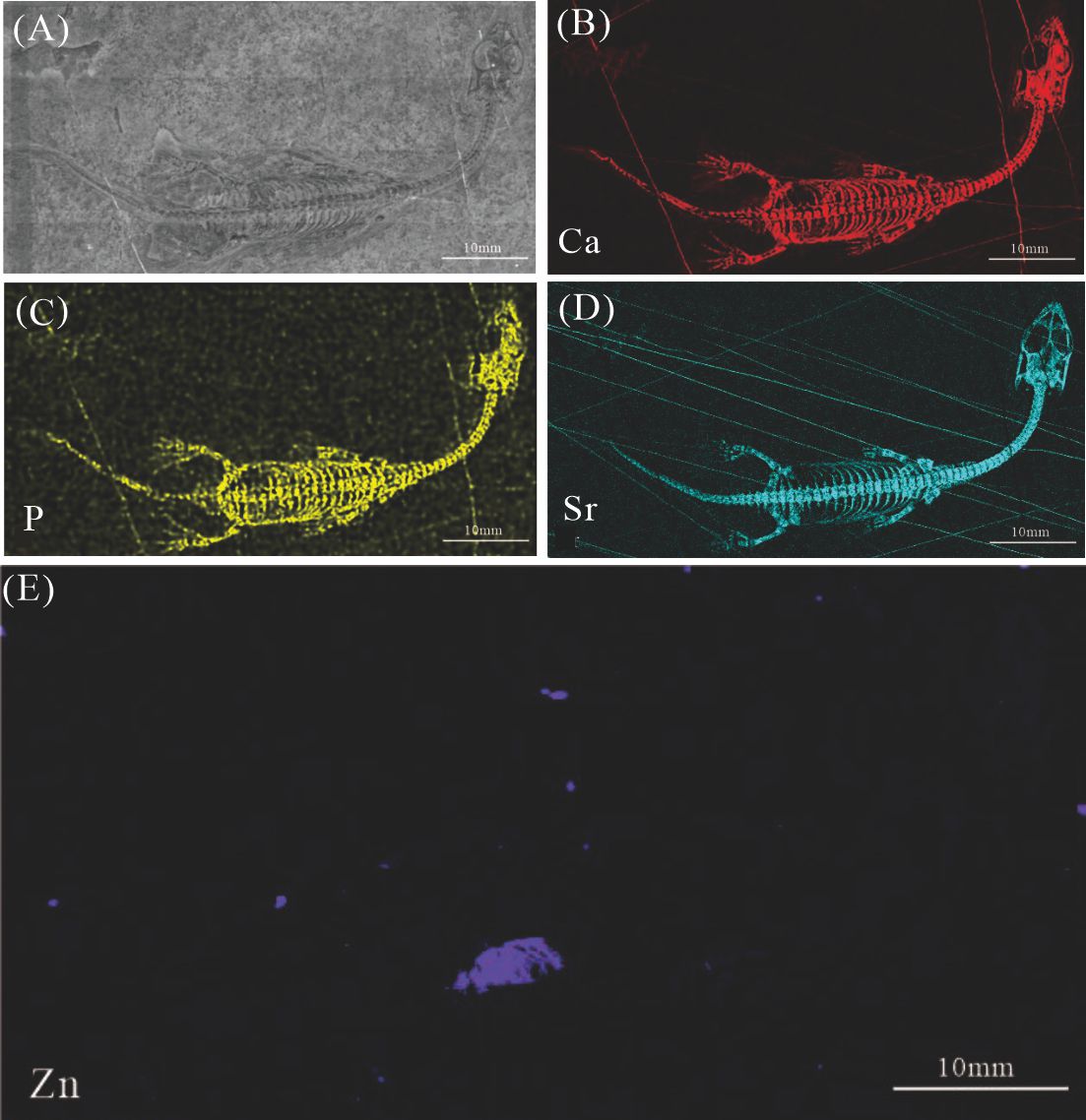

Supplement: Supplemental Information 1 — (A) XNGM WS-31-R22. (B)-(E) Micro-XRF maps (false-color images) of Ca(B), P(C), Sr(D), Zn(E). [file peerj-13-19475-s001.jpg]

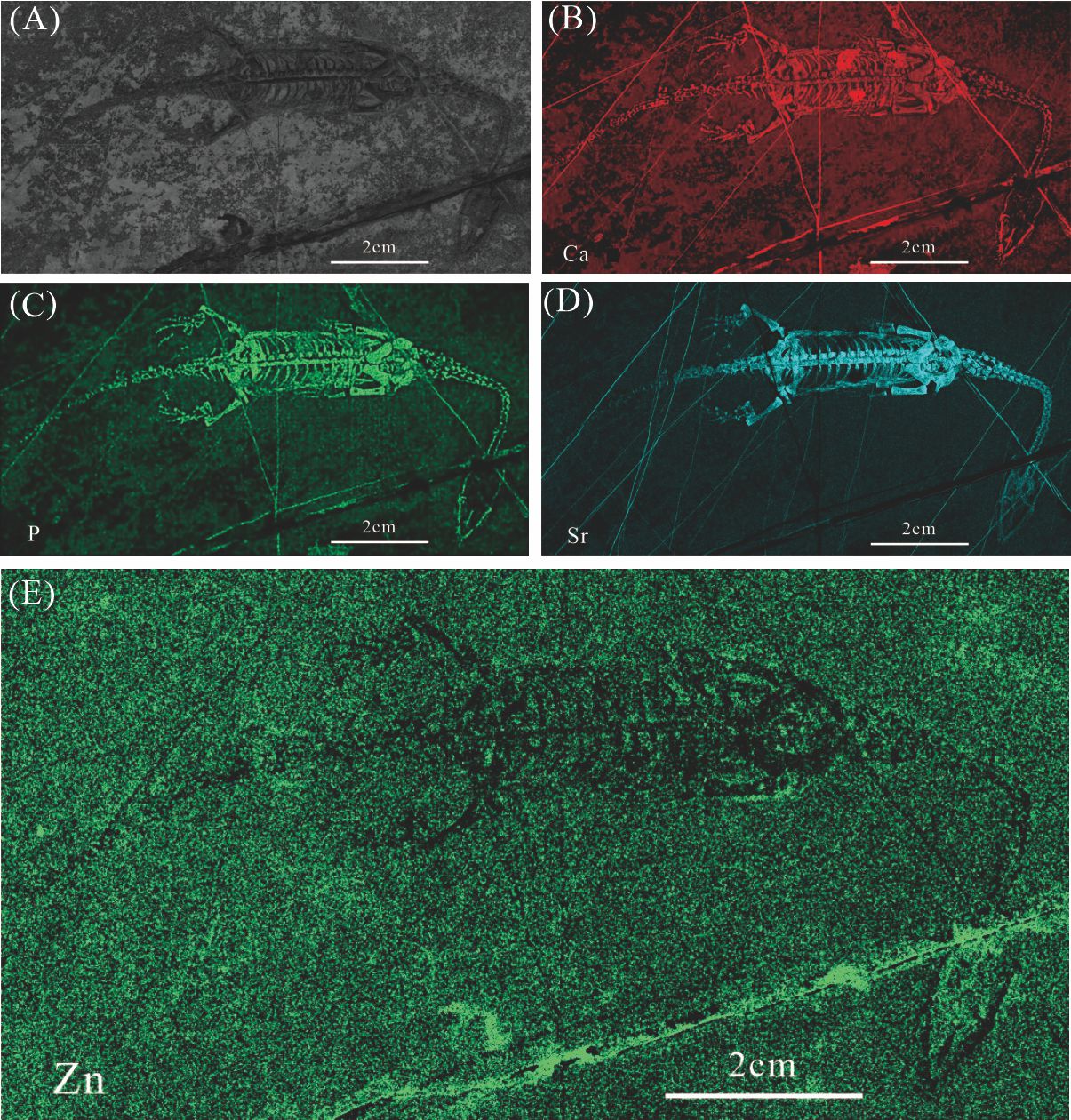

Supplement: Supplemental Information 2 — (A) Photo of GMPKU-P-1154(2). (B)-(E) Micro-XRF maps (false-color images) of Ca(B), P(C), Sr(D), Zn(E). [file peerj-13-19475-s002.jpg]

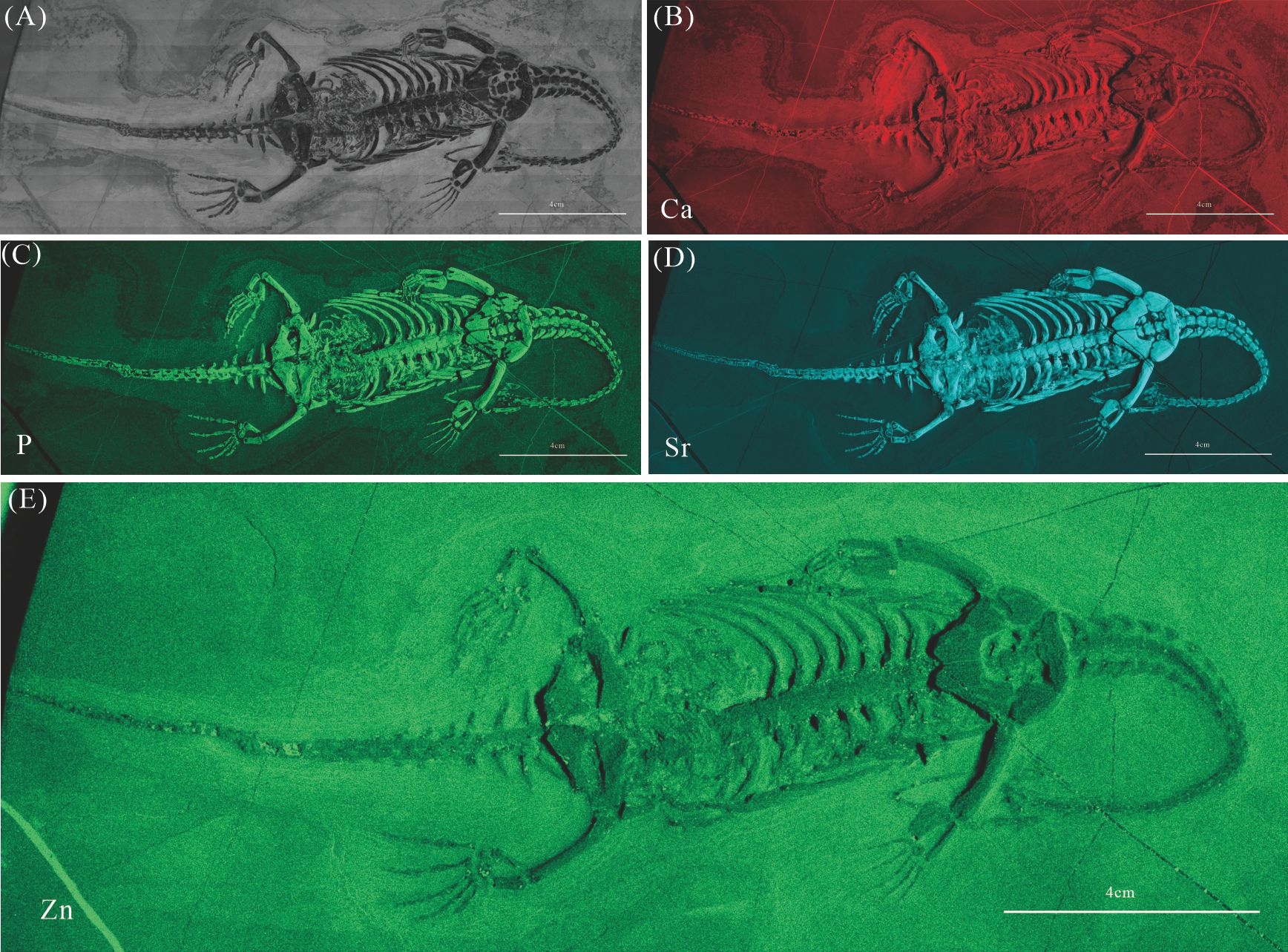

Supplement: Supplemental Information 3 — (A) Photo of GMPKU-P-4317. (B)-(E) Micro-XRF maps (false-color images) of Ca(B), P(C), Sr(D), Zn(E). [file peerj-13-19475-s003.jpg]

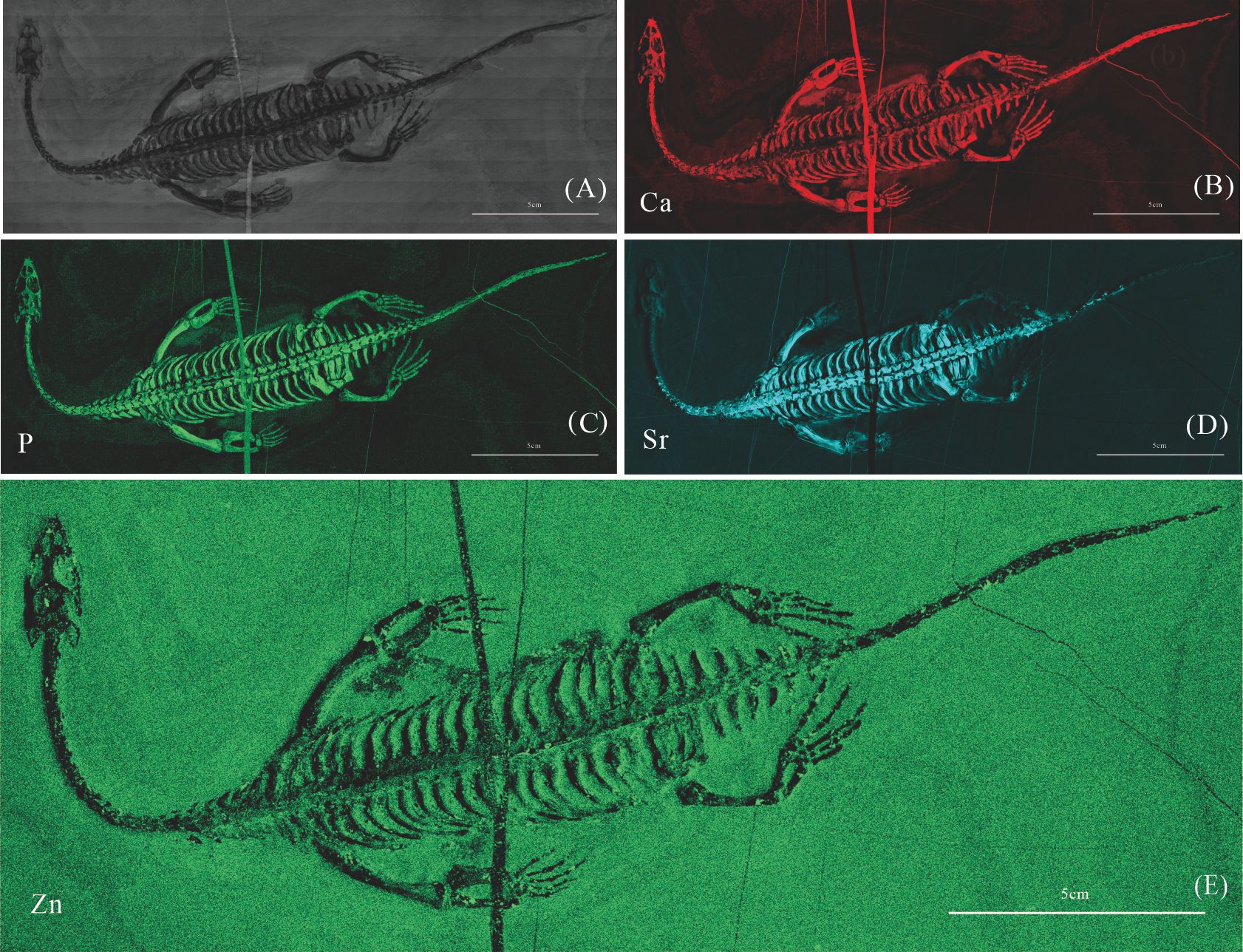

Supplement: Supplemental Information 4 — (A) Photo of GMPKU-P-4318. (B)–(E) Micro-XRF maps (false-color images) of Ca(B), P(C), Sr(D), Zn(E). [file peerj-13-19475-s004.jpg]

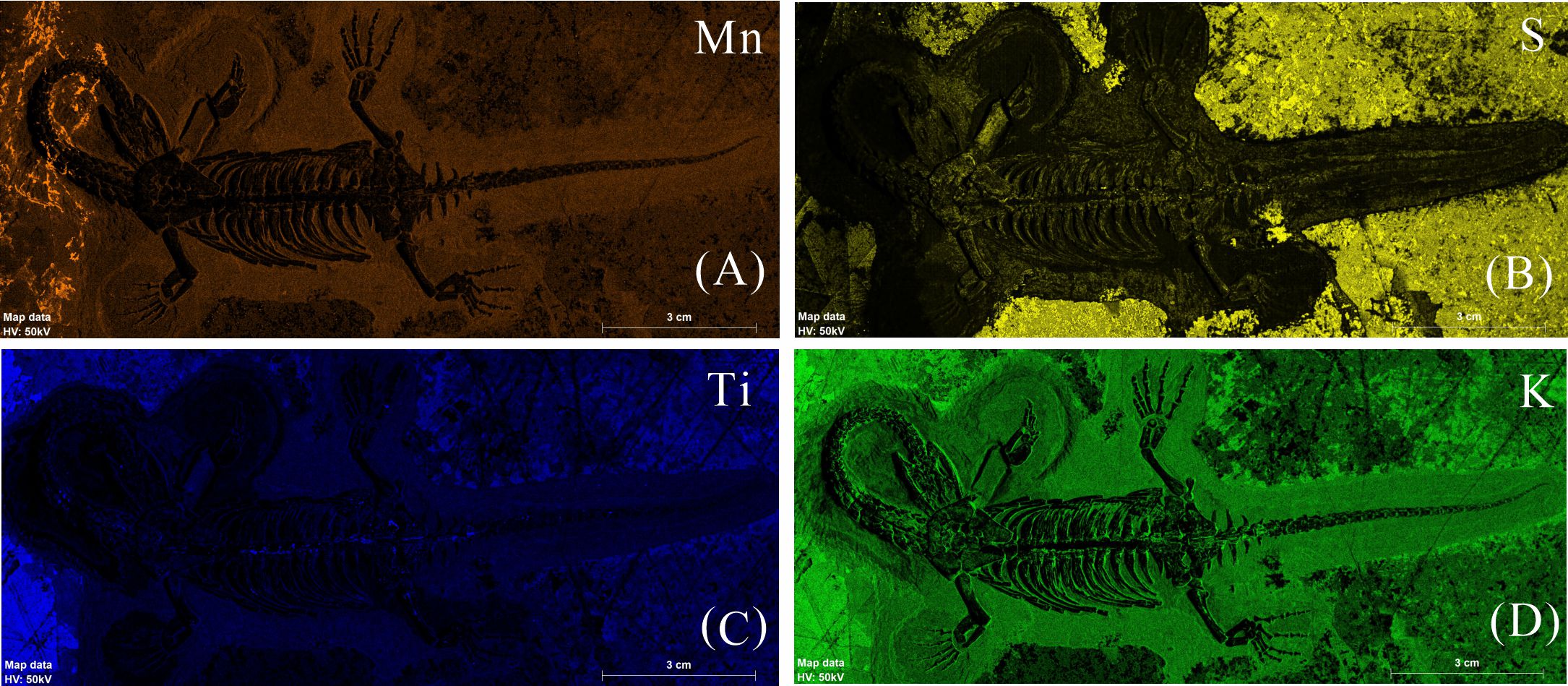

Supplement: Supplemental Information 5 — (A)–(D) Micro-XRF maps (false-color images) of Mn(A), S(B), Ti(C), K(D) [file peerj-13-19475-s005.jpg]
