# Supplementary material for: Skeletal ossification of Middle Triassic pachypleurosaur Keichousaurus hui (Reptilia: Sauropterygia) revealed by zinc distribution
Source: PeerJ. 2025 Jun 18;13:e19475. doi: 10.7717/peerj.19475 (PMC12182052; doi:10.7717/peerj.19475)
Supplement: Supplemental Information 6 [file peerj-13-19475-s006.docx]

**Table S1 The** [**experiment parameter**](javascript:;) **of Micro-XRF in *Keichousaurus hui***

| Specimen Number | High voltage  (kV) | Anode current  (μA) | Width  (mm) | Height  (mm) | Pixel time  (ms/pixel) | Pixel Size  (μm) | Total number of pixel | Measure time |
| --- | --- | --- | --- | --- | --- | --- | --- | --- |
| XNGM WS-31-R22 | 50 | 600 | 62.887 | 35.466 | 10 | 100 | 223295 | 31 min |
| XNGM WS-32-R43 | 50 | 600 | 91.537 | 44.551 | 10 | 100 | 408090 | 56 min |
| GMPKU-P-1154（2） | 50 | 600 | 107.000 | 55.200 | 10 | 100 | 590640 | 1:20 h |
| GMPKU-P-4316 | 50 | 600 | 152.195 | 65.899 | 10 | 50 | 4011992 | 9:14 h |
| GMPKU-P-4317 | 50 | 600 | 202.110 | 73.845 | 10 | 50 | 5970034 | 14:02 h |
| GMPKU-P-4318 | 50 | 600 | 242.060 | 91.947 | 10 | 50 | 8902599 | 20:41 h |
| XNGM WS-32-R18 | 50 | 600 | 224.647 | 123.277 | 10 | 100 | 2769318 | 6:21 h |
| The right hindlimb of GMPKU-P-4316 | 50 | 600 | 22.577 | 13.073 | 15 | 25 | 472269 | 1:37 h |
| The left hindlimb of GMPKU-P-4316 | 50 | 600 | 14.576 | 26.621 | 15 | 25 | 620895 | 2:08 h |
